# Brucellosis-Induced Hemophagocytic Lymphohistiocytosis

**DOI:** 10.7759/cureus.15677

**Published:** 2021-06-16

**Authors:** Jawahar Al Noumani, Ibrahim Al Busaidi, Malak Al Hajri

**Affiliations:** 1 Internal Medicine, Internal Medicine Residency Program, Muscat, OMN; 2 Infectious Diseases Unit, Sultan Qaboos University Hospital, Muscat, OMN

**Keywords:** brucellosis, hemophagocytic lymphohistiocytosis, cytopenia, hepatosplenomegaly, zoonosis

## Abstract

Hemophagocytic lymphohistiocytosis (HLH) is a fatal syndrome, which can be primary or triggered by a systemic disease or an infection. The commonly reported infectious causes of secondary HLH include Epstein-Barr virus (EBV), cytomegalovirus (CMV), mycobacterium, and leishmaniasis among other infections. In this case report, we report a 50-year-old woman with brucellosis-related HLH after presenting with prolonged fever, hepatosplenomegaly, and cytopenia.

## Introduction

Hemophagocytic lymphohistiocytosis (HLH) is a rare life-threatening syndrome manifested by excessive inflammation and tissue destruction due to the absence of normal downregulation by activated macrophages and lymphocytes of the immune system [1]. It can be either primary with a genetic etiology or secondary to a systemic disease or infection [1-4]. The most commonly reported infectious causes are Epstein-Barr virus (EBV), cytomegalovirus (CMV), parvovirus, herpes simplex virus, varicella-zoster virus, measles virus, human herpes virus 8, H1N1 influenza virus, parechovirus, HIV, and SARS-CoV-2 [2,3,5-7]. Other infectious etiologies include mycobacterium, spirochaetes, fungi, and parasites.

HLH is characterized by a triad of prolonged fever, hepatosplenomegaly, and cytopenia [8]. The main diagnostic criteria for HLH are as follows: Familial disease/known genetic defect or clinical and laboratory criteria of five out of eight of the followings: fever, splenomegaly, cytopenia >= 2 cell lines, hypertriglyceridemia and/or hypofibrinogenemia with ferritin >= 500 g/L, sCD 25 (soluble interleukin-2 receptor) >= 2,400 U/mL, decreased or absent natural killer (NK) cell activity or hemophagocytosis in bone marrow (BM), cerebrospinal fluid (CSF), or lymph nodes [9].

Brucellosis is a bacterial zoonotic infection transmitted to humans by contact with infected animals’ fluids or consumption of infected food products. It is characterized by acute or insidious febrile illness with several presentations and complications such as osteoarticular disease, endocarditis, and neurobrucellosis [10]. Brucellosis can be associated with the release of a significant level of inflammatory cytokines, lymphocyte activation, and multi-organ histiocyte infiltration, which result in the development of HLH. The same process causes a rise in the tumor necrosis factor α-levels that inhibit the lipoprotein lipase activity, resulting in elevation of triglyceride levels [11].

We report this case to remind practitioners to consider brucellosis a rare cause of HLH in patients with prolonged fever, hepatosplenomegaly, and pancytopenia.

## Case presentation

A 50-year-old Omani woman presented to the emergency department at Sultan Qaboos University Hospital (Oman) with fever, chills, drenching night sweats, and malaise for two months associated with unintentional weight loss of 11 kilograms over the past few months. The patient reported no respiratory or gastrointestinal symptoms. She had no history of symptoms suggestive of connective tissue diseases and she had no skin rash. She has a significant history of close contact with goats, and she provided regular care to them, but she denied consumption of unpasteurized dairy products or insect bite. There was no travel history or significant sick contact. The patient was previously healthy except for mild iron deficiency anemia for which she received oral iron therapy.

At triaging in the emergency department, she was alert and oriented but looked unwell and diaphoretic. She was febrile (39.6°C) and tachycardic (105 beats per min). Blood pressure 140/74 mmHg, respiratory rate 22 breaths/min, and oxygen saturation was 97% on room air.

General examination showed pallor, but no icterus. She had palpable bilateral anterior cervical lymph nodes, which were non-tender, firm, mobile, and measuring 2 by 3 cm. Abdominal examination revealed soft, non-tender hepatomegaly of 4 cm below the costal margin and palpable spleen tip. The remainder of the physical examinations was normal.

**Figure 1:**
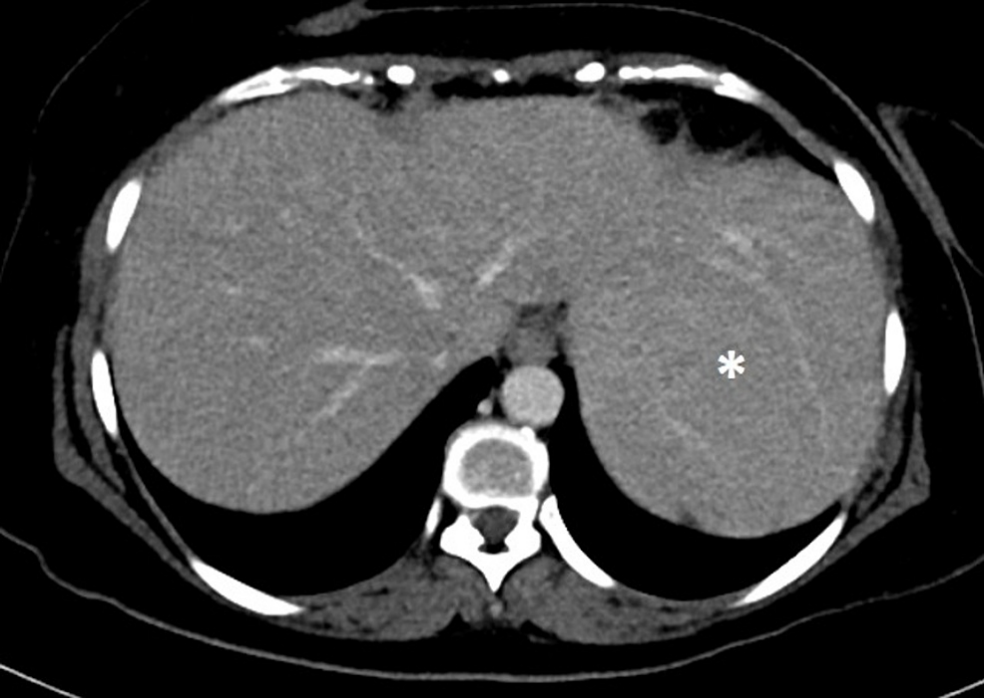
Selected axial image of contrast-enhanced CT mildly enlarged liver mainly the left lobe, which is extending to the lateral side of the left upper quadrant of the abdominal cavity. Left lobe of the liver (*).

**Figure 2:**
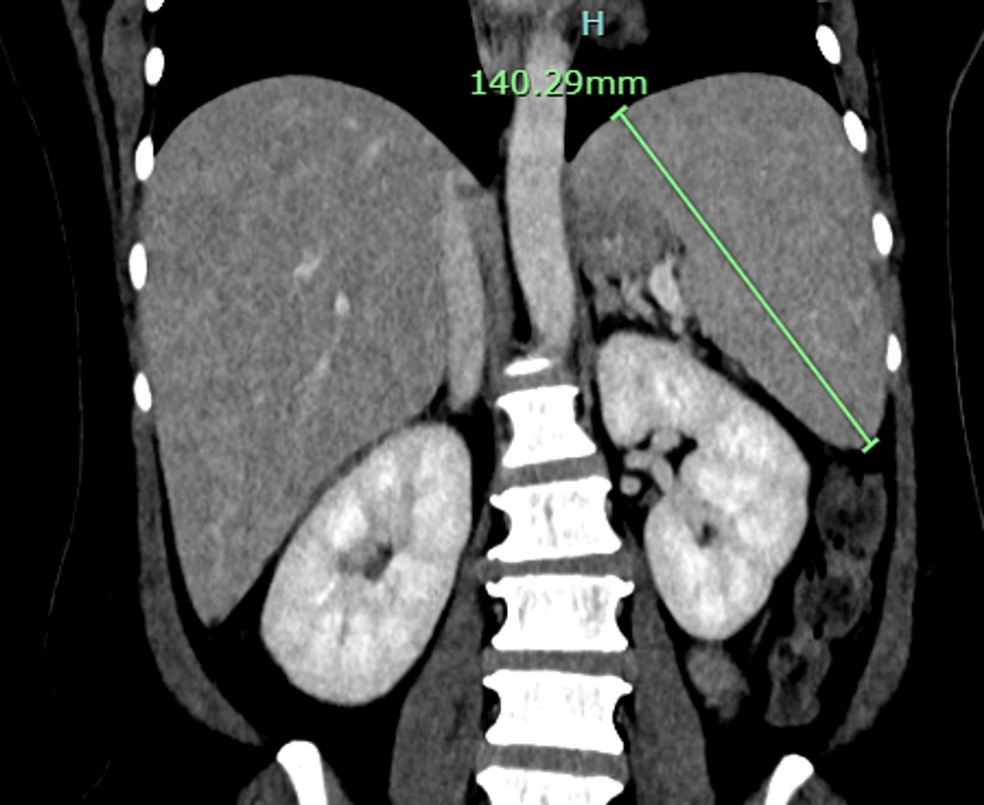
Selected coronal reconstructed image of contrast-enhanced CT abdomen showing mildly enlarged spleen measuring about 14 cm with no focal lesion.

**Figure 3:**
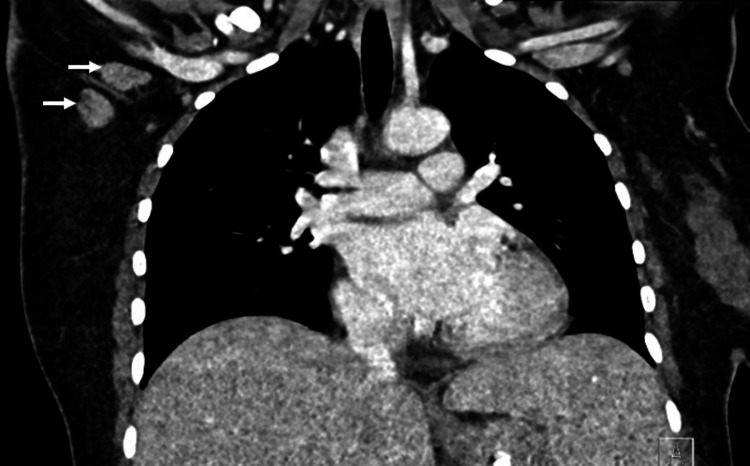
Selected coronal reconstructed image of contrast-enhanced CT chest showing enlarged right side axillary lymph nodes with obliterated fatty hilum (white arrows).

Initial investigations revealed normocytic-normochromic anemia, thrombocytopenia, and leukopenia with moderate lymphopenia. Blood film showed no malignant cells. The coagulation screen was normal. The hepatic function panel showed twofold rise in alanine aminotransferase and sevenfold rise in aspartate aminotransferase with normal alkaline phosphatase albumin and bilirubin. Lactate dehydrogenase was elevated. Ferritin level was very high. Triglyceride level was also elevated (Table 1). Chest x-ray was normal. Cardiac electrocardiography was normal. A full body computerized tomography (CT) scan showed hepatosplenomegaly (Figures 1 and 2) and mildly enlarged right axillary lymph nodes (Figure 3). Blood cultures grew Brucella species after two days of incubation. Transthoracic echocardiography showed no signs of infective endocarditis. In the view of her cytopenia, a bone marrow biopsy was done, and it showed a few histiocytes without any hemophagocytes or malignant cells seen.

**Table 1:** Laboratory investigations on presentation CRP: C-reactive protein; ALT: Alanine aminotransferase; AST: aspartate aminotransferase; ALP: Alkaline phosphatase; LDH:  Lactate dehydrogenase; INR: International normalized ratio

Investigation	Normal range	Results on admission
Hemoglobin	11.0–14.5 g/dL	7.6
White blood cell count	2.4–9.5 x 10^9^/L	1.9
Neutrophils absolute count	1.0–4.8 x 10^9^/L	1.0
Platelet count	150–450 x 10^9^/L	80
Lymphocytes	1.2–3.8 x 10^9^/L	0.8
CRP	0–5 mg/L	180
Ferritin	30-400 μg/L	73,004
ALT	0–33 U/L	79
AST	0–32 U/L	221
ALP	35–104 U/L	92
Triglycerides	0.0–2.3 mmol/L	3.8
LDH	135–214 U/L	1,064
Malaria rapid antigen test and blood film	Negative	Negative twice
Thyroid-stimulating hormone (TSH)	0.27–4.20 mIU/L	2.27
INR	0.9–1.10	1.17

The patient met five components of the diagnostic criteria for HLH and hence a diagnosis of brucellosis-induced HLH was made. The patient was managed with anti-Brucella therapy including intravenous gentamicin (5 mg/kg) for seven days, doxycycline 100 mg twice a day and oral ciprofloxacin 750 mg twice a day replacing rifampicin, which was avoided due to elevated liver enzymes. A few days after starting antibiotic therapy patient defervesced and her constitutional symptoms and oral intake have remarkably improved. Her blood counts completely normalized after three weeks of therapy. She received combined oral anti-Brucella therapy for a total duration of six weeks with a complete clinical recovery.

## Discussion

Brucellosis is a common zoonotic infection with a major public health burden in many parts of the world with a high prevalence in the Middle East and Mediterranean countries. Anemia, thrombocytopenia, leukopenia, and relative lymphocytosis are the most frequently reported hematological abnormalities in patients with brucellosis. Pancytopenia was reported in 2.4% of patients in one study [12]. Hepatosplenomegaly is reported in 37.5% and 56.6% of patients with brucellosis in two different studies [13,14]. This is caused by reticuloendothelial involvement. In our patient, the lymphoproliferative disease was suspected in the view of fever, hepatosplenomegaly, lymphadenopathy, and pancytopenia. However, after the diagnosis of brucellosis was confirmed, the etiology of her profound cytopenia was unclear and this guided for further evaluation. Secondary HLH was suspected and this was later confirmed by meeting the diagnostic criteria despite the bone marrow biopsy did not show major hemophagocytic activity. In our case, brucellosis-induced HLH was suggested by the presence of five of these criteria including fever, splenomegaly, cytopenia (neutropenia, thrombocytopenia, and anemia), hypertriglyceridemia, and hyperferritinemia exceeding 70,000 μg/L.

Brucellosis is rarely reported as a cause of secondary HLH. Innate immune response triggered by toll-like receptors (TLRs) as a response to the infectious agent is one of the mechanisms leading to HLH [8]. Cases of adult patients with brucellosis-related HLH from China, Turkey, and Tunisa have been reported [15-17]. Yaman reported three children with this diagnosis who had a similar presentation to our patient. In addition to standard anti-Brucella therapy, they received intravenous immunoglobulins (IVIG) to manage severe thrombocytopenia despite that evidence for the role of IVIG in the management of HLH is lacking [18]. The treatment of HLH depends on the underlying diseases and the severity of the symptoms. In infection-related HLH, appropriate antimicrobial therapy and supportive care is the mainstay of therapy [8]. However, in severe cases and organ failure, an additional short course of corticosteroids and/or intravenous immunoglobulin therapy should be considered to control hypercytokinemia [8]. Our patient had a complete clinical recovery and resolution of cytopenia by treating the brucellosis without the need for immunomodulatory therapy.

## Conclusions

HLH is a rare but potently fatal complication of brucellosis that should be considered in patients with high-risk exposure to animals or consumption of unpasteurized dairy products and clinical presentation of febrile illness, organomegaly, and cytopenia. The diagnosis is supported by profound hyperferritinemia and elevated triglycerides level. Appropriate anti-Brucella therapy and supportive care is the mainstay of therapy. The addition of immunomodulators should be considered in patients with severe disease and organ failure.
